# Moderate plant–soil feedbacks have small effects on the biodiversity–productivity relationship: A field experiment

**DOI:** 10.1002/ece3.7819

**Published:** 2021-08-10

**Authors:** Josephine Grenzer, Andrew Kulmatiski, Leslie Forero, Anne Ebeling, Nico Eisenhauer, Jeanette Norton

**Affiliations:** ^1^ Department of Wildland Resources and the Ecology Center Utah State University Logan UT USA; ^2^ Institute of Ecology and Evolution University of Jena Jena Germany; ^3^ German Centre for Integrative Biodiversity Research (iDiv) Halle‐Jena‐Leipzig Leipzig Germany; ^4^ Institute of Biology University of Leipzig Leipzig Germany; ^5^ Department of Plant, Soils and Climate Utah State University Logan UT USA

**Keywords:** aboveground–belowground interactions, biodiversity–ecosystem functioning, biomass, dominance, plant community model, plant identity

## Abstract

Plant–soil feedback (PSF) has gained attention as a mechanism promoting plant growth and coexistence. However, most PSF research has measured monoculture growth in greenhouse conditions. Translating PSFs into effects on plant growth in field communities remains an important frontier for PSF research. Using a 4‐year, factorial field experiment in Jena, Germany, we measured the growth of nine grassland species on soils conditioned by each of the target species (i.e., 72 PSFs). Plant community models were parameterized with or without these PSF effects, and model predictions were compared to plant biomass production in diversity–productivity experiments. Plants created soils that changed subsequent plant biomass by 40%. However, because they were both positive and negative, the average PSF effect was 14% less growth on “home” than on “away” soils. Nine‐species plant communities produced 29 to 37% more biomass for polycultures than for monocultures due primarily to selection effects. With or without PSF, plant community models predicted 28%–29% more biomass for polycultures than for monocultures, again due primarily to selection effects. *Synthesis*: Despite causing 40% changes in plant biomass, PSFs had little effect on model predictions of plant community biomass across a range of species richness. While somewhat surprising, a lack of a PSF effect was appropriate in this site because species richness effects in this study were caused by selection effects and not complementarity effects (PSFs are a complementarity mechanism). Our plant community models helped us describe several reasons that even large PSF may not affect plant productivity. Notably, we found that dominant species demonstrated small PSF, suggesting there may be selective pressure for plants to create neutral PSF. Broadly, testing PSFs in plant communities in field conditions provided a more realistic understanding of how PSFs affect plant growth in communities in the context of other species traits.

## Graphical abstract

Plant–soil feedbacks have gained attention as a mechanism of plant growth and coexistence, yet most plant–soil feedback research continues to be performed in greenhouse conditions. At the end of a 4‐year field experiment, plants created soils that changed subsequent plant growth by 37%, yet these relatively large effects had little effect on plant community growth because some feedbacks were positive and others negative and because feedbacks for the dominant species were small. Research highlights the need to test the consequences of plant–soil feedbacks in plant communities.

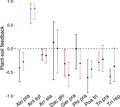

## 1 INTRODUCTION

Plant–soil feedbacks (PSFs) have gained attention over the past 25 years as a potential mechanism of plant growth and coexistence (Bever, 1994; van der Putten et al., 2013). Yet, most PSF research has been performed using plant monocultures in greenhouse conditions (Forero et al., 2019; Kulmatiski et al., 2008). Recent work suggests that these greenhouse experiments provide little insight into plant growth in field communities (Forero et al., 2019; Reinhart et al., 2021). There remains, therefore, a need to better understand the role of PSF in plant communities in the field (Kulmatiski & Kardol, 2008; Lekberg et al., 2018).

One robust trait of plant communities that may be, at least in part, explained by PSF is that productivity tends to increase with diversity (Kulmatiski et al., 2012; Tilman et al., 2001; Weisser et al., 2017). It has long been thought that the positive diversity–productivity relationship can be explained because species extract resources in different times or places (i.e., niche partitioning or complementarity; Hector et al., 1999; Loreau & Hector, 2001; Tilman et al., 1997). This mechanism can explain both species coexistence and why more diverse communities are more productive (i.e., because they more fully exploit resource space; Barry et al., 2019; Loreau et al., 2001). However, resource complementarity has been found to be insufficient to explain either the extent of, or variation in, diversity–productivity relationships (Barry et al., 2019; Hector et al., 1999; Schnitzer et al., 2011). For example, despite overall positive diversity effects, some species and communities underyield in diversity–productivity experiments (Hector et al., 1999). As a result, there has been interest in discovering additional mechanisms that contribute to diversity–productivity relationships (Eisenhauer et al., 2012; Loreau et al., 2001).

Selection effects and disease accumulation have been suggested as additional mechanisms underlying positive diversity–productivity relationships (Loreau & Hector, 2001; Maron et al., 2011; Schnitzer et al., 2011). Selection effects occur if species with “selected” traits disproportionately affect mixtures at the expense of other species (Loreau & Hector, 2001; Roscher et al., 2008). Disease accumulation can cause overyielding if species‐specific diseases accumulate and suppress plant growth more in low‐diversity communities than in high‐diversity communities (i.e., pathogen dilution; Maron et al., 2011; Mommer et al., 2018; Schnitzer et al., 2011). However, neither selection effects nor disease accumulation are likely to explain the wide range of overyielding and underyielding observed in diversity–productivity experiments (Hector et al., 1999, Kulmatiski et al., 2012).

Plant–soil feedbacks have been suggested as a mechanism that can explain both underyielding and overyielding (Kulmatiski et al., 2012). PSF describes a process in which plants change soil conditions, which can then affect further plant growth (conspecific or heterospecific; Bever, 2003; Hamilton & Frank, 2001; Wardle et al., 2004). These effects are often attributed to soil microbial communities (Ehrenfeld et al., 2005, Ke & Wan, 2020, Reynolds et al., 2003), but they can also result from changes to soil chemistry (Ehrenfeld et al., 2005, Smith‐Ramesh & Reynolds, 2017), soil structure (Kyle et al., 2007), and soil animals (Eisenhauer, Reich, & Scheu, 2012).

Disease accumulation is one component of PSF that results in negative PSFs and can be expected to cause overyielding (Maron et al., 2011; Mommer et al., 2018; Schnitzer et al., 2011). Conversely, symbiont accumulation is another component of PSF, potentially resulting in a positive PSF. For example, a plant that accumulates species‐specific symbionts can be expected to benefit more from those symbionts in a dense monoculture than in a diverse community (Kulmatiski et al., 2012). The role of plant mutualists in soil has been reported to affect plant community performance (Latz et al., 2012; Wagg et al., 2011) and suggested to codetermine selection and complementarity effects (Eisenhauer, 2011; Eisenhauer et al., 2012). However, positive PSF can also occur when a species' growth is suppressed by soils cultivated by a different species (e.g., allelopathy; van der Putten et al., 2016). In either case, species with positive PSFs can be expected to be more productive in monoculture than in polyculture (i.e., they underyield; Kulmatiski et al., 2012).

While conceptually appealing, the magnitude of PSF effects in plant communities remains poorly understood for several reasons. Across the literature, roughly two‐thirds of plants create negative PSFs, and one‐third create positive PSFs (Cortois et al., 2016; Kulmatiski et al., 2008; Lekberg et al., 2018; van der Putten et al., 2016). However, most PSF research has been performed in the greenhouse and greenhouse‐derived PSFs have been found to be larger than and uncorrelated with field‐derived PSFs (Forero et al., 2019; Kulmatiski et al., 2008; Schittko et al., 2016). Further, most PSF research has measured PSFs without explicitly testing the role of the PSFs in plant mixtures (Ke & Wan, 2020; Kulmatiski et al., 2012; van der Putten et al., 2013). As a result, it is not known whether PSFs affect species coexistence or community productivity or whether PSFs are overwhelmed by other factors related to plant growth such as competitive interactions, herbivory, or intrinsic growth rates (Heinze & Joshi, 2018; Kulmatiski et al., 2011; Lekberg et al., 2018; Reinhart et al., 2021).

The overarching goal of this study was to test the role of PSFs in the diversity–productivity relationship. We established paired PSF and diversity–productivity experiments with mesic grassland species in Jena, Germany. Working in this site allowed us to test PSF effects in two diversity–productivity experiments (Roscher et al., 2016). We report PSF values and their relationship to competitive ability, but the emphasis of this paper was to test whether or not PSF, as measured in monocultures, can improve predictions of plant growth in communities (i.e., plant growth in diversity–productivity experiments). To do this, a suite of plant community growth models was parameterized with or without PSF data, and model predictions were compared to plant biomass in 2‐year‐old and three‐year‐old plant communities. Consistent with modeling and greenhouse experiments, we predicted that PSF effects would improve model predictions of plant community productivity because (a) PSFs would be predominantly negative and explain overyielding and (b) positive PSFs would occur and contribute to underyielding (Kulmatiski et al., 2012; Maron et al., 2011; Schnitzer et al., 2011).

## 2 METHODS

### 2.1 Site

In 2015, we established PSF and diversity–productivity experiments in the Jena Experiment field site on the floodplain of the Saale River, Jena, Germany, with eutric fluvisols (5–33 g C kg^−1^ and 1.0–2.7 g N/kg soil; Roscher et al., 2004; Weisser et al., 2017). Long‐term mean annual temperature and precipitation at the site are 9.8°C and 544 mm (2002–2018), respectively, and during the experiment (2015–2018), mean annual temperature and precipitation were 10.4°C and 499 mm, respectively (Kolle, 2020). The first and last years of the experiment (2015 and 2018) were drier than average, 459 mm and 395 mm, respectively, while 2017 was wetter than average (615 mm).

### 2.2 Field experiments

The PSF experiment followed a two‐phase, factorial bio‐assay approach (Bever, 1994; Brinkman et al., 2010). This design is considered one of the most robust PSF experimental designs, because it measures plant growth in the field, on each soil type without mixing soils (Kulmatiski & Kardol, 2008; Rinella & Reinhart, 2018). In Phase I, monocultures of each plant species were grown for two growing seasons to create soils with a known plant cultivation history (nine soil treatments). Plants were then removed. In Phase 2, each plant species was grown for two growing seasons on replicate plots with each plant cultivation history (Bever, 1994; Brinkman et al., 2010; Rinella & Reinhart, 2018). At the same time, we performed a diversity–productivity experiment that replicated an experiment installed in 2002 by Roscher et al. (2004). In both experiments, plant communities with 1, 2, 3, 4, 6, and 9 species were grown. Plant species included five grasses: *Alopecurus pratensis, Arrhenatherum elatius, Dactylis glomerata, Phleum pratense, and Poa trivialis*; two tall herbs: *Anthriscus sylvestris and Geranium pratense*; and two legumes: *Trifolium pratense and Trifolium repens* (Roscher et al., 2004).

In fall 2014, a 75 × 22 m area was mowed, sprayed with glyphosate herbicide (Roundup^®^ 0.045% v/v pelargonic acid; Evergreen Garden Care Österreich GmbH, Salzburg), and tilled using several passes to 30 cm with an agricultural cultivator. Herbicide and tillage treatments may affect PSF, but were the same across all treatments (Helander et al., 2018). For the PSF experiment, a grid with 1,251 plots was created. To isolate each 35 cm wide by 75‐cm‐long plot from each other, a 10 cm wide by 35‐cm‐deep trench was dug around the outside of plots, and a custom‐made, flat‐bladed shovel was used to slice soils between plots to allow insertion of a 35‐cm‐deep root barrier (RootBlock® 1 mm high‐density polyethylene; GreenMax, Netherlands). Each of the nine target species was randomly assigned to 139 replicate plots. In March 2015, seeds (4 g/m^2^) were applied by hand for one species in each plot. Prior to seeding, seeds of *Anthriscus sylvestris* were stored at −20°C for 2 weeks (Roscher et al., 2004). Due to poor establishment, *Anthriscus sylvestris* and *Geranium pratense* plots were reseeded in October 2015 with 2,000 germinating seeds [7.5 g/m^2^ and 28.3 g/m^2^, respectively; germination rates based on Roscher et al. (2004)]. Nontarget species were removed by hand at least three times each growing season, and, consistent with other experiments at the site, aboveground biomass was harvested and removed each spring and fall as is typical for hay meadows in Central Europe (Roscher et al., 2004).

Phase 1 ended in September 2016, when standing biomass was removed and plots were treated with herbicide to prevent resprouting. Roughly 2 weeks later, plots were hand‐tilled to further prevent resprouting of Phase 1 species. Plots were randomly assigned so that each plant species was grown in 14 replicate plots that had grown the same species in Phase 1 (i.e., “home” soils) and 15 replicate plots that had grown each of the other species in the experiment in Phase 1 (i.e., “away” soils). Five replicate “home” plots remained unseeded to assess the extent of resprouting growth in “home” plots. It is important to distinguish new growth from resprouting, because resprouting growth would result in inappropriately positive PSF values. Mean resprouting growth varied in these control plots varied from 0 to 32 g/m^2^ and was removed from final biomass estimates in “home” plots. On 15 March 2017, 2,000 pure live seeds m^−2^ were applied by hand to each PSF plot. In October 2017 and June‐July 2018, biomass from Phase 2 plots was clipped to 5 cm above soil surface by hand, dried to constant weight at 70°C, and weighed.

The 2014 diversity–productivity experiment included 223 plots (1.5 m by 1.5 m), also lined with root barriers. Monocultures were replicated three times (9 species × 3 replicates = 27 plots). Each of the 91 plant communities grown in the 2002 experiment was grown in one plot (91 plots; Appendix S1; Roscher et al., 2004). Additionally, six randomly selected communities of two, three, four, and six species mixtures were replicated in four plots (4 species richness levels * 6 communities* 4 replicates = 96). Communities with all nine target species were replicated in nine plots. In March 2015, target seed mixtures with 2,000 pure live seeds per m^2^, equally distributed among species, were applied by hand to each plot. Again, aboveground biomass was clipped to 5 cm above soil surface in November 2015 and June and October 2016 and 2017. A subsample of 0.1 m^2^ per plot was sorted by plant species, dried at 70°C for 3 days, and weighed. As in the PSF experiment, nontarget species were removed by hand at least three times per year from 2015 to 2017.

### 2.3 Calculating plant–soil feedbacks

Plant–soil feedbacks were calculated as the difference of growth on “home” and “away” soils divided by the maximum of “home” and “away” soils and were used to parameterize plant community models. This calculation is comparable to the log response ratio, but has the advantage that its values are bound by −1 and +1 and are readily interpreted as the proportional change in growth (Brinkman et al., 2010; Kulmatiski et al., 2012). Soil‐level PSF values describe the growth of each plant species on each of eight “away” soils resulting in 72 soil‐level PSF values (i.e., eight values for each of nine species). Species‐level PSF values describe the growth of each species across the other eight soil types resulting in nine PSF values. In both cases, PSF values and associated 95% confidence intervals were calculated using 2000 bootstrapped biomass on home and away samples (Kulmatiski et al., 2017; Schittko et al., 2016). PSF values with confidence intervals that do not overlap zero are considered significant (i.e., positive or negative, as appropriate).

### 2.4 Calculating relative competition intensity (RCI)

A goal of this research was to test whether or not PSFs improve predictions of plant growth in communities where other factors such as a plant's competitive ability are also important (Lekberg et al., 2018). To better understand how PSF may interact with a plant's competitive ability, we correlated PSFs with the relative competition index (RCI, Weigelt & Jolliffe, 2003) where RCI = (monoculture biomass−twice the two‐species mixture biomass)/monoculture biomass. A low RCI indicates higher biomass production of a species in two‐species mixtures than in monocultures (strong competitor). RCI was calculated for the 2002 and 2014 diversity–productivity experiments.

### 2.5 Simulating plant growth in communities

A suite of species‐specific, individual plant growth models was parameterized with or without PSF data (Kulmatiski et al., 2016). The models and modeling approach generally follow that of Kulmatiski et al. (2016), but briefly, the foundation of these models is logistic growth equations (Equation 1). In addition to the effects of intrinsic plant growth rates r, total plant biomass in the community P, and a carrying capacity Κ, plant growth is also a function of soil conditions σ. Plants are assumed to change soil conditions as they grow with different rates on different soil conditions (Bever, 1994; Kulmatiski et al., 2011). Without PSF effects, plant growth rates are the same across all soil conditions. Plants can “compete” indirectly through carrying capacity, but competition coefficients were not included. Models were parameterized with different carrying capacities, data from different years (input data), and with different values for “neutral” soils (defined below) to produce a suite of simulations. Average biomass predictions from this suite of model parameterizations are reported. The goal of this modeling is to simulate relatively short‐term plant growth in the field experiment and not to determine equilibrium species abundances (Feng et al., 2020; Kulmatiski et al., 2011).

To include PSF effects in these models, each plant species i conditions soil j and therefore has a soil‐specific growth rate r_i,j_. The biomass of plant species i at time t (P_i,t_) depends on its growth rate at t (ri,t) and is limited by either community‐level carrying capacity Κ alone (Equation 1) or additional species‐level carrying capacity κ_i_ (Equation 2). At the community‐level, Κ simulates interspecific competition, but “competitive strength” is only defined by growth rates: Κ is defined as the maximum biomass a community can achieve, whereas at the species‐level, κ_i_ simulates intraspecific competition: κ_i_ is defined as the maximum biomass a species can achieve. The time‐ and plant‐specific growth rate r_i,t_ represents the summed product of soil‐specific growth rates r_i,j_ and the proportion of soil at time (σ_j,t_; Equations 3 and 4). Assuming gradual change of soil conditions as plants grow, we estimate growth rate on unconditioned soil (“neutral” growth rate, ν_i_) and set the abundance of neutral soil to one (100%) at t = 0 (Equation 3). While plants grow, neutral soil is subsequently replaced by conditioned soil.

(1) $$P_{\text{i,t}+1}=P_{\text{i,t}}+P_{\text{i,t}}r_{\text{i,t}}\left(1‐\Sigma_{i=1}^{N}P_{\text{i,t}}/K\right)$$

(2) $$P_{\text{i,t}+1}=P_{\text{i,t}}+P_{\text{i,t}}r_{\text{i,t}}\left(1‐\Sigma_{i=1}^{N}P_{\text{i,t}}/K‐P_{\text{i,t}}/\kappa_{i}\right)$$

(3) $$\sigma_{\text{j,t}}=P_{\text{i,t}}/K$$

(4) $$r_{\text{i,t}}=\Sigma_{\text{i = 1}}^{N}\sigma_{\text{i,t}}r_{\text{i,j}}+\left(1‐\Sigma_{i=1}^{N}\sigma_{\text{i,t}}\right)\nu_{i}$$

Growth rates on conditioned soil (r_i,j_) were derived from plant biomass on PSF plots (input data) in 2017 and/or 2018 (Kulmatiski et al., 2011). “Neutral” growth rates (ν_i_) were set to be growth rates on “home”, “away”, or across all PSF plots. Growth rates were calculated from final biomass on different soil types. For example, for P_i,j_, r_i,t_ = (P_i,j_/P_0_)^1/T^−1, where T = 52 time steps and P_0_ = 4 g/m^2^. Carrying capacities were defined as the mean ± two standard deviations of total plant biomass (Κ) in diversity–productivity plots or plant species biomass (κ) across all PSF plots. Models were run for three 52‐time step iterations (t). To simulate harvest, each 53rd time step, plant biomass was set to 1% of P_i,t_. Simulations were performed in R (R Core Development Team, 2015). Two models (Equations 1 and 2), three sources of input data (2017 only, 2018 only, or 2017 then 2018), and three “neutral” growth rates (home, away, all plots) produced 18 null and 18 PSF model simulations.

### 2.6 Dissecting mechanisms driving the diversity–productivity relationship

We estimated the net biodiversity effect based on calculations proposed by Loreau and Hector (2001), which estimate the yield increase ΔY of a plant community compared to the combined performance of plant species in monocultures. We further used equations to partition the net biodiversity effect (ΔY) into selection and complementarity effects (Loreau & Hector, 2001).

### 2.7 Statistics

To describe species richness effects, we fit random intercept (linear mixed) and linear mixed models in R (R Core Development Team, 2015) using lme4 (Bates et al.,l., 2015; Schielzeth & Forstmeier, 2009; Schmid et al., 2017). Due to low establishment in 2015, we analyzed species biomass data by plot and year (i.e., the sum of spring and fall harvests as g dry mass m^‐2^) for 2016 and 2017. Analyses followed those of Roscher et al. (2008). Maximum likelihood was used to find the “best” random model among random intercepts such as species composition (com), year, and their interaction (Roscher et al., 2008; Roscher et al., 2016). The effects of block (soil gradient; Huston & McBride, 2002; Weisser et al., 2017) and the interaction of block and Year (block:Year) were included as random intercepts for the 2002 experiment. From these random intercept models with no fixed effects, we extracted fixed effects of interest (Schmid et al., 2017). The contrast of monoculture and polyculture (MP) and the linear contrast of species richness (SR) were fixed effects (MP+SR). When analyzing mechanisms of the diversity–productivity relationship (selection and complementarity), the model was fit without the contrast of mono‐ and polycultures. Mixed models were fit with maximum likelihood to derive statistical significance of fixed effects from likelihood‐ratio tests (*Χ* ^2^; Roscher et al., 2016). To avoid pseudoreplication, replicate plots were averaged prior to analyses so that the diversity–productivity dataset had 300 samples (100 species compositions × 3 years). To compare species richness effects between experiments and simulations, a second random intercept model was used with the fixed effects model: data+SR+data:SR and random intercept model: com+com:Year, where data are the data source (i.e., observed, PSF, or null model predictions).

## 3 RESULTS

### 3.1 Plant–soil feedback

**FIGURE 1:**
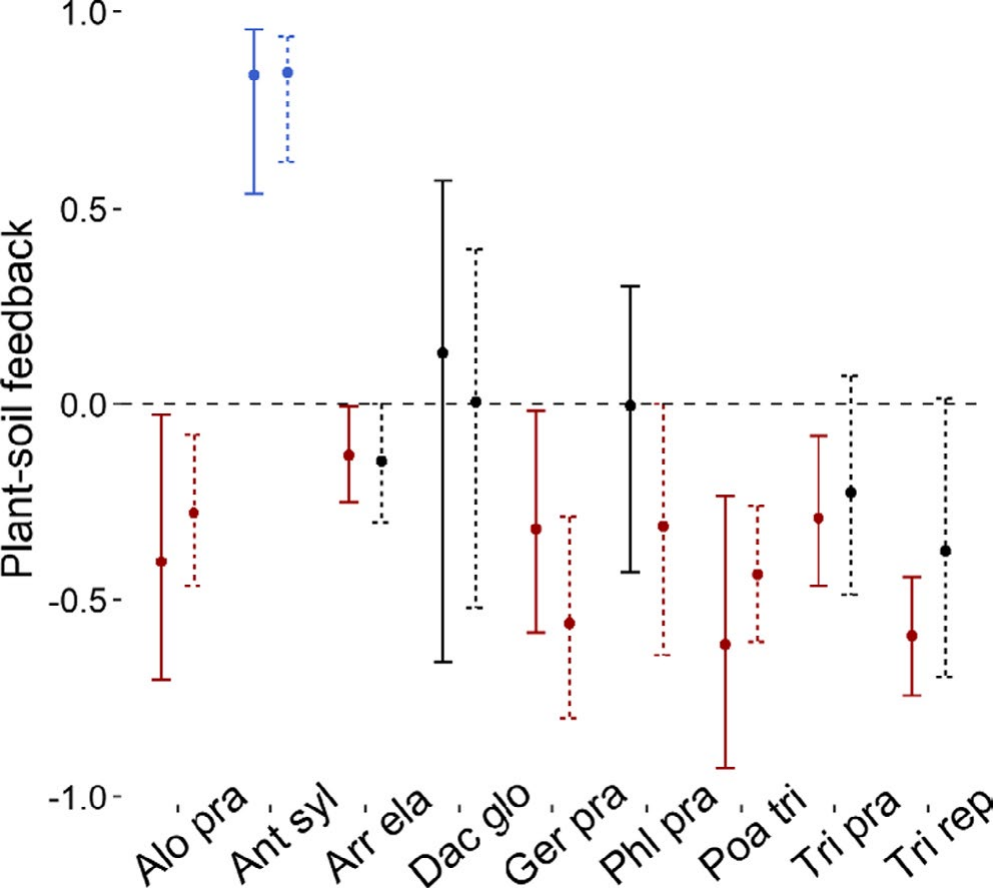
Species‐level plant–soil feedback (PSF) for nine grassland species, Jena, Germany. Values represent the mean and variation in PSF observed across the eight other soil types in the experiment. Positive values indicate the proportion to which a plant grows better on “home” than on “away” soils. Negative values indicate the proportion to which a plant grows better on “away” than on “home” soils. Solid and dotted lines are from fall 2017 and fall 2018, respectively. Error bars represent 95% confidence intervals from bootstrapped values. Red, black, and blue values represent negative, neutral, and positive PSFs, respectively. Species abbreviations on the x‐axis: five grass species: *Alopecurus pratensis* (Alo pra), *Arrhenatherum elatius* (Arr ela), *Dactylis glomerata* (Dac glo), *Phleum pratense* (Phl pra)*, and Poa trivialis* (Poa tri)*;* two tall herbs: *Anthriscus sylvestris* (Ant syl) *and Geranium pratense* (Ger pra)*;* and two legumes: *Trifolium pratense* (Tri pra) *and Trifolium repens* (Tri rep)

For plant species, there were six negative, one positive, and two neutral PSFs in 2017 (Figure 1). The mean absolute value of these PSFs was 0.37 (the 95% confidence interval [CI_95_] was 0.16 to 0.58). In other words, plants created soils that changed subsequent plant growth 37%. The arithmetic mean value of species‐level PSF was −0.15 (CI_95_ −0.50 to 0.19). In 2018, five species demonstrated non‐neutral PSF (Figure 1), though neither absolute (0.35) nor arithmetic (−0.17) PSF values differed between 2017 and 2018 (t_abs_ = 0.25; t_art_ = 0.17; *p* > .05, DF = 8; *paired t test;* Figure 1).

**FIGURE 2:**
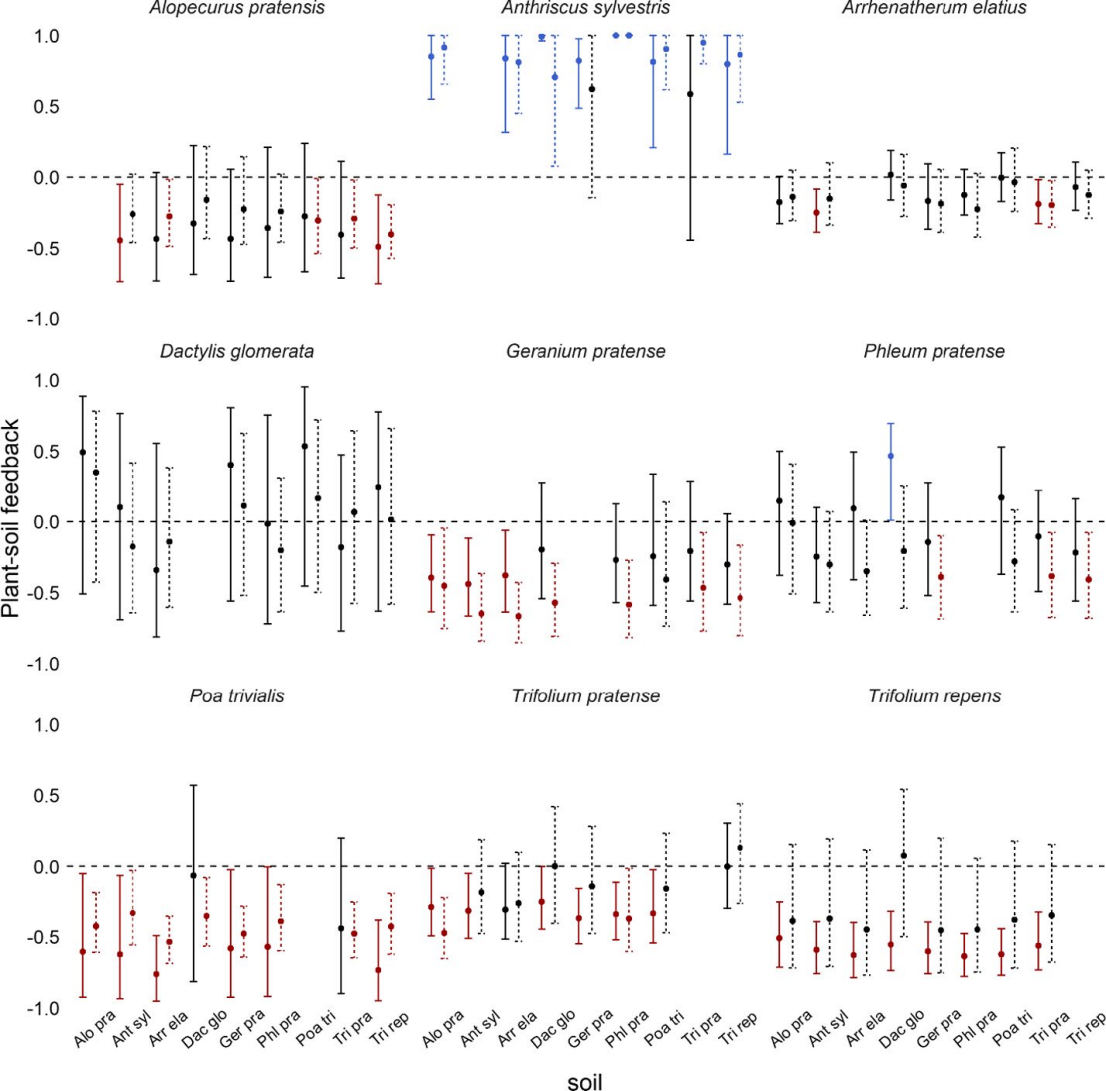
Soil‐level plant–soil feedback (PSF). Each panel shows the PSFs for a plant species across eight soil types. Soil types are defined by the plant species that cultivated them. Solid and dotted lines are from fall 2017 and fall 2018, respectively. Each value derived from target plant biomass in 14 plots with “home” soils and 15 plots with “away” soils. Error bars represent 95% confidence intervals of bootstrapped values. Red, black, and blue values represent negative, neutral, and positive PSFs, respectively. Species abbreviations listed in Figure 1

Twenty‐seven of 72 factorial PSFs were negative and eight were positive in 2017 (Figure 2). The mean of absolute soil‐level PSFs was 0.40 (CI_95_ = 0.34 to 0.45), while the arithmetic mean value was −0.14 (CI_95_ = −0.24 to −0.03). In 2018, absolute values (0.36, CI_95_ = 0.31 to 0.42), and the arithmetic mean (−0.15, CI_95_ = −0.25 to −0.05) were similar to and did not differ from 2017 values (t_abs_ = 1.50; t_art_ = 0.44; *p* > .05, *df* = 71; *paired t* *test*) though only 13 of 72 factorial PSFs differed from zero in 2018 (Figure 2).

**FIGURE 3:**
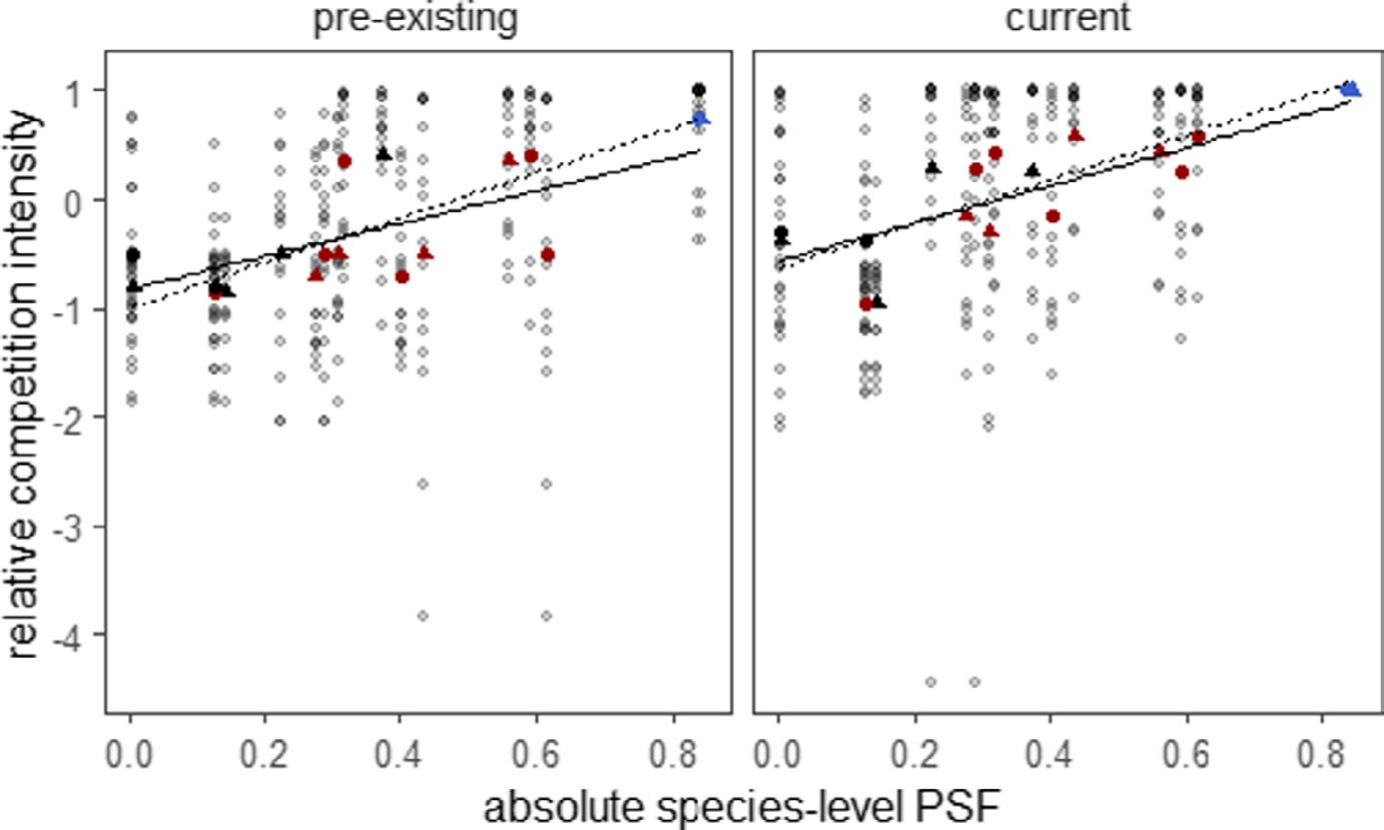
Correlation of the absolute value of species‐level plant–soil feedback (PSF) and relative competition intensity (RCI). The positive slope indicated that competitive species were associated with small PSF values and poor competitors were associated with large PSF values. A low RCI indicates greater biomass in two‐species communities than would be expected from monocultures (i.e., a strong competitor). Solid lines and filled circles from 2017, and dashed lines and filled triangles from 2018. 2014 data from 2016 to 2017, and 2002 data from 2003 to 2004. All correlations were significant (*p* < .05), *R* ^2^ = 0.11–0.23. Red, black, and blue values represent negative, neutral, and positive PSFs, respectively

Competitive species demonstrated small PSF values and poor competitors demonstrated large positive or negative PSF values (Figure 3). More specifically, mean species RCI values from both 2002 and 2014 experiments were correlated with absolute PSF values from 2017 (2002: Pearson's *R* = .690, *p* < .01:2014: Pearson's *R* = .819, *p* < .01) and 2018 (2002: Pearson's *R*, *p* < .01; 2014: Pearson's *R* = .826, *p* < .01).

### 3.2 Observed and predicted biodiversity effects

**FIGURE 4:**
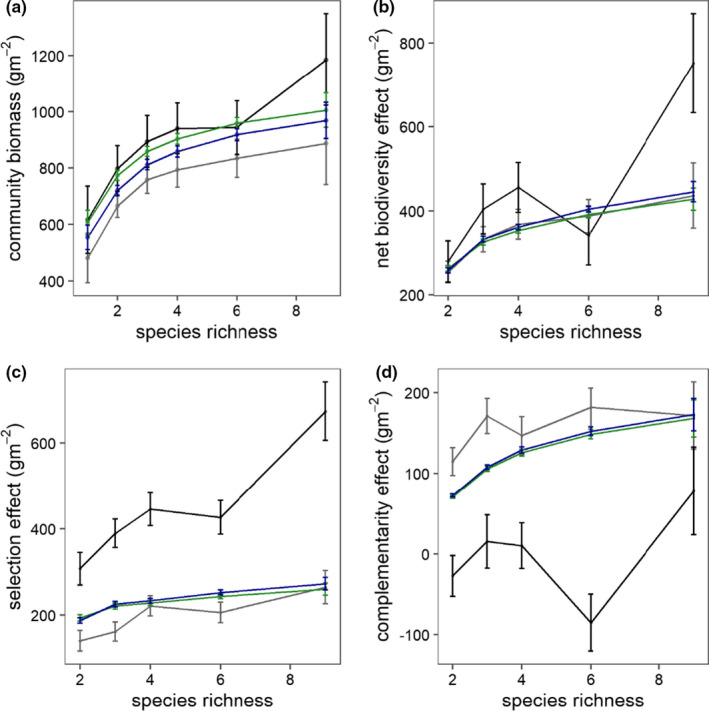
Observed and predicted species richness effects for (a) community biomass, (b) net biodiversity effects, (c) selection effects, and (d) complementarity effects. Data from 2014 (2016–2017) and 2002 experiments (2003–2004; Roscher et al., 2004) shown in black and gray, respectively. Null and PSF model predictions shown in green and blue, respectively. Standard error bars shown represent error from replicate field plots. Statistical analyses in Tables S1 and S2

Polycultures produced 40% (2002) and 55% (2014) more biomass than monocultures, respectively (Figure 4; Table S1). In both experiments, selection effects were greater than complementarity effects (Figure 4; Table S1). Selection effects increased with species richness in the 2002 experiment. Complementarity effects were unrelated to species richness in either experiment (Figure 4; Table S1). Between experiments, community biomass, net biodiversity, and selection effects were greater in the 2014 than in the 2002 experiment. Complementarity effects were smaller in the 2014 experiment than in the 2002 experiment (Figure 4; Table S1). Predictions of biodiversity effects never differed between PSF and null models (Table S2). Null and PSF model predictions did not differ from observed biomass or net biodiversity, but predicted selection effects were smaller and predicted complementarity effects were larger than observed in the 2014 experiment (Figure 4; Table S2).

In regression analysis of community biomass, models with and without PSFs explained 30% and 28% of the variation in the biomass of communities in the 2014 experiment. Both models explained 8% of variation in the 2002 experiment (Table S3). Similarly, for species biomass, models with and without PSF explained 40% and 42% of the variation in the 2014 experiment and 36% and 38%, respectively, of the variation in the 2002 experiment.

### 3.3 Predicted and observed species abundance

**FIGURE 5:**
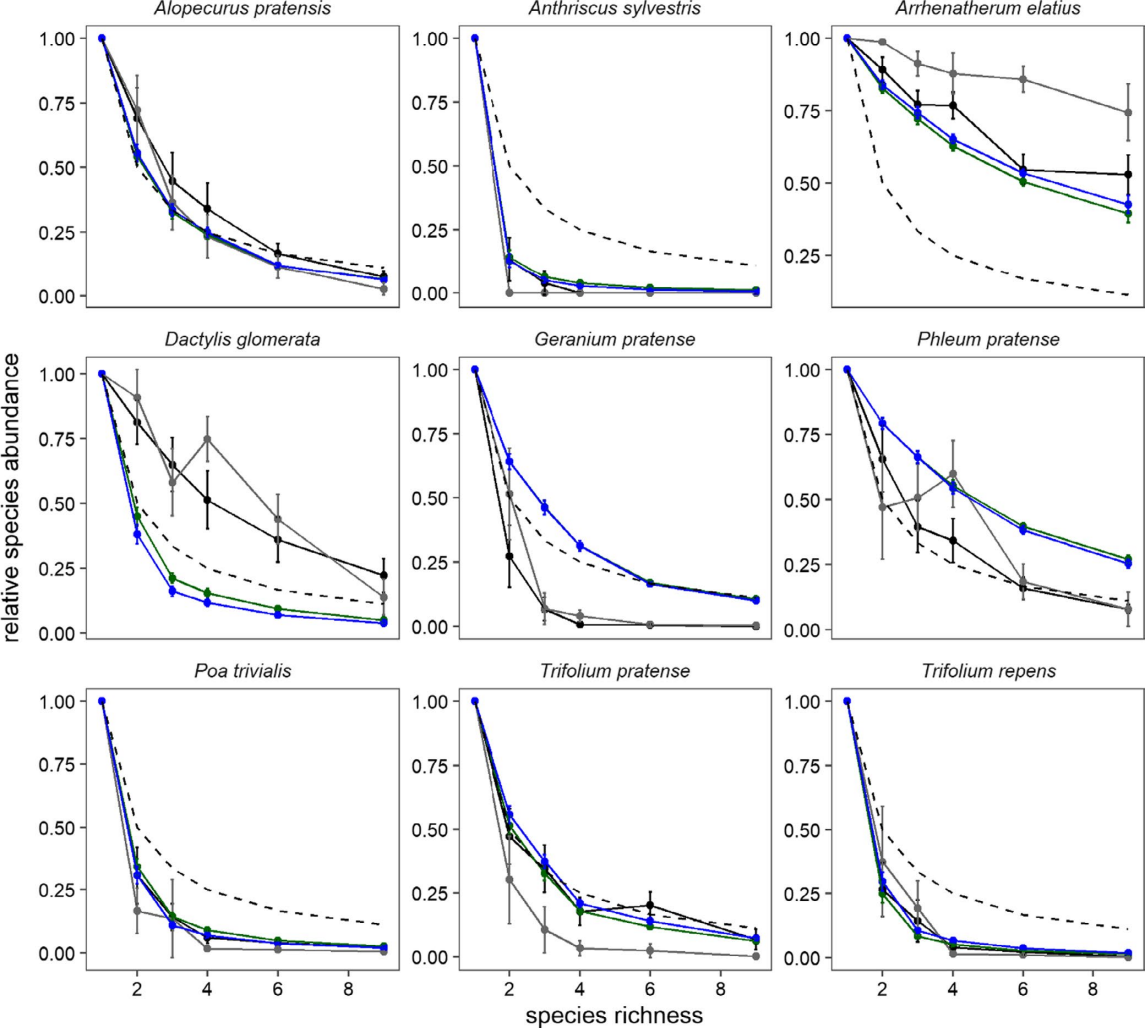
Observed and predicted relative abundance for nine target species. The dashed line represents a default prediction of plant growth which was calculated as 1/species richness. Observed and modeled data (solid lines) located above the dashed line indicate that a species was more productive in communities than would be predicted (i.e., overyield). Observed data from a 2014 experiment (2016–2017) and a 2002 experiment (2003–2004, Roscher et al., 2004) shown in black and gray, respectively. Null and PSF model predictions shown in green and blue, respectively. Statistical analyses in Table S4

In the 2002 and 2014 experiments, communities were dominated by three grass species (*A. elatius*, *D. glomerata*, and *P. pratense*; Table S4; Figure 5). Models with and without PSF correctly predicted dominance by *A. elatius* and *P. pratense*, but neither model predicted *D. glomerata dominance*. When present, *A. elatius* represented 74% to 90% of biomass and increased community biomass (Figure 5; Table S1). The second and third most abundant species both attained similar relative biomass in both experiments. In the two experiments, *D. glomerata* and *P. pratense* represented 58% to 61% and 40% to 44% of biomass in communities in which they were found, respectively (Figure 5). Because *D. glomerata* performed poorly in Phase 2, models underestimated its relative biomass as 19% to 23% without and with PSF effects, respectively.

## 4 DISCUSSION

Because most PSF research continues to be performed on plant monocultures in greenhouse conditions, the extent to which PSFs affect plant communities in the field remains unclear (Crawford et al., 2019; Forero et al., 2019; Ke & Wan, 2020; Reinhart et al., 2021). Our factorial experiment provided unusually comprehensive information about PSFs in the field. We measured all possible PSFs for nine species and found that plants, on average, created soils that changed subsequent plant growth by 40% (CI_95_ = 34 to 45). However, because plants realized both positive and negative PSFs, the average effect was that plants grew 14% (CI_95_ = −24 to −3) less on home than on away soils. While most PSF studies simply measure PSFs, we also tested the effect of these PSFs in plant communities. Despite causing 40% changes in plant biomass, PSFs had little effect on model predictions of plant community biomass across a range of species richness. While somewhat surprising, a lack of a PSF effect was appropriate because species richness effects at the study site were caused by selection effects and not complementarity effects (PSFs are a complementarity mechanism).

Plant–soil feedbacks had little effect on model predictions for several reasons. First, even though the absolute value of PSFs was reasonably large, the average PSF effect was small because some PSFs were positive, while others were negative. Second, PSFs for the two dominant plant species were small (−0.14 to 0.12). Third, because PSFs were, on average, smaller than differences in intrinsic growth rates (40% versus 193%), they were unlikely to change competitive outcomes between species (Kulmatiski et al., 2016; Lekberg et al., 2018). Finally, *A. elatius* dominated across all species richness levels. As a result, “away” soils had little effect on *A. elatius* growth regardless of species richness. Broadly, our results demonstrated that large PSF values alone are not sufficient to explain plant species coexistence or the diversity–productivity relationship at this site. In fact, overyielding at the site was caused primarily by selection effects, so complementarity effects of any kind (e.g., niche partitioning or PSF) were unimportant.

Our plant community growth simulation models correctly predicted relatively large selection effects and small complementarity effects. However, these models also underestimated the total selection effect. It is likely that including competition coefficients into PSF models would have increased *A. elatius* growth and selection effects. Alternatively, it is possible that these selection effects will decrease over time and that model predictions may be more consistent with longer‐term patterns of plant community growth (Fargione et al., 2007).

Results do not exclude a role for PSF as a mechanism of species coexistence and productivity in the long‐term particularly at other sites with larger complementarity effects, rather results highlight that PSF effects must be considered in the context of other factors affecting plant growth such as intrinsic growth rates, competition, or facilitation (Jing et al., 2015; Crawford et al., 2019; Lekberg et al., 2018).

Results provide an important contribution to the literature because PSFs were measured in field conditions during a 4‐year experiment. While this approach was expected to provide more realistic insight into how PSF is likely to affect plant growth in communities on the landscape, several experimental artifacts may affect interpretation. Plots were treated with herbicide and tilled during the experiment. These treatments are likely to affect plant growth, competition among species and PSF, but importantly these treatments were consistent across all plots. Climate variability (e.g., winter, dry years) and herbivory in the field plots may also have affected PSF. It was not possible to assess the role of all these interacting effects, but, because these factors were largely held constant across treatments, results provided important insight into the extent of PSFs in field conditions.

### 4.1 The curious case of plant–soil feedbacks and the dominant species

We predicted that negative PSFs would cause overyielding because soil pathogens would be “diluted” in diverse communities relative to monocultures (Kulmatiski et al., 2012; Maron et al., 2011; Schnitzer et al., 2011). However, *A. elatius* was such a dominant species that it maintained at least 75% relative biomass across all species richness levels in the 2014 diversity–productivity experiment. From a PSF perspective, an important consequence of this dominance is that *A. elatius* effectively only grew on “home” soils. Therefore, *A. elatius* never benefited from pathogen dilution on “away” soils. Research examining potential PSF effects “in vitro” often assumes that species are competitively equivalent (Bever, 2003; Kulmatiski et al., 2011). Performing this experiment in field conditions helps refocus the role of PSF in the context of strong competitive imbalances among species which are common in field conditions (Crawford et al., 2019; Lekberg et al., 2018).

### 4.2 Are neutral PSFs a successful strategy?

In addition to primarily growing on “self” soils, the dominant species *A. elatius* realized a small PSF with little variability within or across soil treatments (Figure 2). It is possible that small PSFs and small variability covary. It is reasonable to expect that, for a plant species to dominate in many communities, it will grow well across soil treatments and, therefore, demonstrate small and consistent PSFs. In contrast, plant species with large positive PSFs may have difficulty establishing in “away” soils, while species with a large negative PSF may have difficulty attaining large growth on “home” soils (Levine et al., 2006). Our results suggest that there may be a selective pressure to maintain neutral PSFs with low variability to dominate plant communities. Consistent with this idea, we found that competitive species were associated with small PSF values (Figure 3), while subdominant species demonstrated large positive and large negative PSF. This perspective may help explain why PSFs often show weak correlations with landscape abundance (Reinhart et al., 2021, but see Mangan et al., 2010; Kulmatiski et al., 2017).

There is also a statistical reason that dominant species may demonstrate small PSFs. It is more likely that plant species with small growth will realize large proportional changes in growth (Pfisterer & Schmid, 2002). For example, a plant species that can grow to 50 g/m^2^ on “home” soils can easily be imagined growing to 0 or 200 g/m^2^ on “away” soils, resulting in PSFs of 1.0 and −0.75, respectively. However, it is essentially impossible for plant species to grow to 1,000 g/m^2^ on “home” soils and 4,000 g/m^2^ on “away” soils because 4,000 g/m^2^ is beyond carrying capacity in grasslands. As a result, subdominant species are more likely to have large PSFs than dominant species. We are not aware of other studies suggesting these ideas and this is likely because PSF experiments rarely use the large factorial experiments needed to examine PSFs for many species across soil types (Rinella & Reinhart, 2018). It is certainly possible that, in some systems, dominant species may be associated with large positive or even large negative PSF, but here we provide explanations for why we observed small, consistent PSFs for the dominant species.

### 4.3 Diversity–productivity relationships

Species richness effects were similar to other biodiversity experiments in more mesic sites (Cardinale et al., 2011; Hector et al., 1999). However, the mechanisms driving this response differed between sites and years. Complementarity effects have been found to be larger in nutrient‐poor sites as well as in the 2002 relative to the 2014 experiments at this site. In the 2002 experiment, polyculture biomass was driven by selection (21% of monoculture biomass) and complementarity (14%) effects. In the 2014 experiment, overyielding was largely explained by selection effects (43%) and countered by negative complementarity effects (−20%). *A. elatius* was more dominant in the 2014 than in the 2002 experiment (Figure 5; Clark et al., 2020). Community productivity in the Jena Experiment varies widely among years due to different environmental conditions (Weisser et al., 2017), so it is likely that climate or other environmental conditions that differed between the two studies also caused greater dominance effects in the 2014 experiment (Guimarães‐Steinicke et al., 2019; Marquard et al., 2009). A large flooding event in 2013 may have increased *A. elatius* growth by increasing nutrient availability (Wright et al., 2015), simultaneously reducing complementarity effects (Roscher et al., 2016). *A. elatius* is strongly competitive for light and nitrogen, so greater seeding rates in the 2014 experiment may have exaggerated asymmetric competitive effects (Lorentzen et al., 2008; Roscher et al., 2008). It is interesting to note that, even though the mechanisms differed, the net biodiversity effect was similar in the new and old experiments. It is unclear, however, how selection, complementarity, and PSF effects will continue to change over longer time periods that are important for the long‐term development of plant communities (Fargione et al., 2007).

It has been suggested that PSFs will intensify competitive effects in nutrient‐rich conditions and strengthen facilitative effects in nutrient‐poor conditions (Bever, 2003; Lekberg et al., 2018). Consistent with this idea, we found that PSFs were more negative, and competitive effects (selection effects) were larger in the 2014 experiment, performed at a mesic, nutrient‐rich site relative to a similar recent study performed at a drier and nutrient‐poor site (Forero, 2021). Both absolute (0.40 versus 0.27) and average (−0.14 versus. 0.10) PSFs were larger at the nutrient‐rich versus. nutrient‐poor site, respectively (Forero, 2021). Further, overyielding was smaller at the nutrient‐rich site than at the nutrient‐poor site (Craven et al., 2016; Forero, 2021). Larger PSFs and competitive effects in nutrient‐rich conditions provide a potential explanation for why the strength and trajectory of biodiversity–ecosystem functioning relationships over time differ between more and less fertile soils (Eisenhauer et al., 2019; Guerrero‐Ramírez et al., 2019; Ratcliffe et al., 2017).

### 4.4 Species‐level versus soil‐level PSFs

Because sample sizes increase exponentially as species are added to factorial PSF experiments, most studies measure PSFs for one to a few target species (Smith‐Ramesh & Reynolds, 2017; Van der Putten et al., 2013). By measuring all 72 potential PSFs for nine species, this study provided unusually comprehensive insights into how PSFs vary among soil conditioned by different species. For the most part, PSFs were consistent among soil treatments. It is not unreasonable to expect PSFs to vary widely across differently conditioned soils (Bezemer et al., 2006; Rinella & Reinhart, 2018; Smith‐Ramesh & Reynolds, 2017). For example, a plant species may grow well on a soil conditioned by a N‐fixing species and poorly on a soil conditioned by an early‐successional species that accumulated a large pool of generalist soil pathogens (Chapin et al., 1994; Van der Putten et al., 2013). However, we observed only one species that demonstrated both positive and negative PSF on different soil treatments (*P. pratense*). The fact that PSF values were consistent across soil treatments suggests that PSFs in this system are determined primarily by growth on “home” soil.

## 5 CONCLUSION

To affect species coexistence, or to have large effects on plant community productivity, PSFs must be large relative to differences in intrinsic growth rates among species (Crawford et al., 2019; Ke & Wan, 2020; Lekberg et al., 2018). While PSFs changed plant growth within plant species by 40%, this effect was smaller than differences in growth among species, and the dominant plant species demonstrated small PSFs in our experiment. The lack of an effect of PSFs on plant communities was surprising, but appropriate because complementarity effects did not contribute to overyielding observed in the 2014 diversity–productivity experiment. Our results demonstrate that species identity and composition of the plant communities can determine whether PSFs are important to plant community growth: Large PSFs for subdominant species and small PSFs for dominant species will cause small overall effects on plant community productivity. Our results also highlight a potential connection between PSFs and competitive ability (Lekberg et al., 2018; Petermann et al., 2008). More specifically, there may be selective pressure for species to produce both small PSFs and large competitive ability to dominate. Results provide an important but uncommon perspective on the role of PSF in plant communities in field conditions.

## CONFLICT OF INTEREST

None declared.

## AUTHOR CONTRIBUTIONS

**Josephine Grenzer:** Conceptualization (lead); Data curation (lead); Formal analysis (lead); Investigation (lead); Methodology (lead); Project administration (lead); Resources (lead); Software (lead); Supervision (lead); Validation (lead); Visualization (lead); Writing‐original draft (lead); Writing‐review & editing (lead). **Andrew Kulmatiski:** Conceptualization (lead); Data curation (lead); Formal analysis (lead); Funding acquisition (lead); Investigation (lead); Methodology (lead); Project administration (lead); Resources (lead); Software (equal); Supervision (equal); Validation (equal); Visualization (equal); Writing‐original draft (equal); Writing‐review & editing (equal). **Leslie Forero:** Conceptualization (equal); Methodology (equal); Writing‐review & editing (equal). **Anne Ebeling:** Data curation (equal); Funding acquisition (equal); Investigation (equal); Methodology (equal); Project administration (equal); Resources (equal); Supervision (equal); Writing‐review & editing (equal). **Nico Eisenhauer:** Formal analysis (equal); Funding acquisition (equal); Investigation (equal); Methodology (equal); Resources (equal); Supervision (equal); Writing‐review & editing (equal). **Jeanette Norton:** Conceptualization (equal); Data curation (equal); Formal analysis (equal); Funding acquisition (equal); Investigation (equal); Methodology (equal); Project administration (equal); Resources (equal); Supervision (equal); Validation (equal); Visualization (equal); Writing‐review & editing (equal).

## DATA AVAILABILITY STATEMENT

All data used in the manuscript are publicly available at the USU Digital Commons (https://doi.org/10.26078/52k0‐jr94, https://doi.org/10.15142/T3XM19).

## Supporting information

App S1
