# Supplementary material for: Moderate plant–soil feedbacks have small effects on the biodiversity–productivity relationship: A field experiment
Source: Ecol Evol. 2021 Aug 10;11(17):11651–63. doi: 10.1002/ece3.7819 (PMC8427583; doi:10.1002/ece3.7819)
Supplement: Supplementary file 1 — App S1 [file ECE3-11-11651-s001.docx]

Table 1 Summary of linear mixed model analysis of observed and predicted community biomass. We fit random intercept models to estimate strength of species richness and species presence. The fixed effect model MP + SR was tested against the random intercepts com and Year·com (interaction), where MP = contrast of mono- and polycultures, SR = linear contrast of species richness, com = species composition, Year = Year of measurement (repeated measurements). Additional random intercept were included for (b) pre-existing experiment (block=soil gradient; Huston & McBride, 2002; Weisser et al., 2017) and Null (c) and PSF (d) models (model, basic model equations and input data used for general parametrization). Reported standard error of random effects derive from random intercept model with fixed effects model: MP+SR. Statistics for species each derive from separate model fits, were absence/presence (AP) of each species was included (MP+SR+AP). Estimates and standard error (SE) are given in gm-2. Models were fit in R (R Core Development Team, 2015) with lmer (lme4, Bates et al., 2015), statistics were obtained with anova, Type III Analysis of Variance with Satterthwaite's method.

|  | **(a)     current experiment** | | | | **(b)    pre-existing experiment** | | | | **(c)     Null model** | | | | **(d)    PSF model** | | | |
| --- | --- | --- | --- | --- | --- | --- | --- | --- | --- | --- | --- | --- | --- | --- | --- | --- |
|  | est | SE | *2* | *P* | est | SE | *2* | *P* | est | SE | *2* | *P* | est | SE | *2* | *P* | |
| **(A)** |  |  |  |  |  |  |  |  |  |  |  |  |  |  |  |  | |
| (intercept) | 835 | 32 | 203 | 0.000 | 717 | 43 | 19 | 0.000 | 827 | 54 | 50 | 0.000 | 778 | 52 | 50 | 0.000 | |
| MP | 248 | 96 | 6 | 0.011 | 264 | 56 | 20 | 0.000 | 242 | 59 | 16 | 0.000 | 246 | 66 | 13 | 0.000 | |
| SR | 53 | 20 | 7 | 0.009 | 41 | 11 | 14 | 0.000 | 45 | 11 | 15 | 0.000 | 48 | 13 | 13 | 0.000 | |
| *A. pratense* | -52 | 67 | 1 | 0.418 | -12 | 34 | 0 | 0.722 | -30 | 36 | 1 | 0.396 | -32 | 40 | 1 | 0.415 | |
| *A. sylvestris* | -143 | 68 | 4 | 0.035 | -99 | 32 | 9 | 0.003 | -119 | 34 | 12 | 0.000 | -129 | 38 | 11 | 0.001 | |
| *A. elatius* | 353 | 56 | 34 | 0.000 | 180 | 29 | 34 | 0.000 | 195 | 30 | 37 | 0.000 | 235 | 32 | 44 | 0.000 | |
| *D. glomeratis* | 125 | 68 | 3 | 0.063 | 33 | 34 | 1 | 0.317 | -32 | 36 | 1 | 0.365 | -66 | 40 | 3 | 0.093 | |
| *G. pratensis* | -36 | 68 | 0 | 0.589 | -64 | 33 | 4 | 0.054 | 38 | 36 | 1 | 0.284 | 36 | 40 | 1 | 0.364 | |
| *P. pratense* | 51 | 68 | 1 | 0.436 | -1 | 34 | 0 | 0.983 | 157 | 32 | 22 | 0.000 | 172 | 36 | 21 | 0.000 | |
| *P. trivialis* | -115 | 67 | 3 | 0.080 | -80 | 33 | 6 | 0.016 | -79 | 35 | 5 | 0.022 | -101 | 39 | 7 | 0.009 | |
| *T. pratense* | -20 | 69 | 0 | 0.775 | 71 | 33 | 5 | 0.032 | -21 | 36 | 0 | 0.542 | -8 | 40 | 0 | 0.838 | |
| *T. repens* | -183 | 67 | 7 | 0.006 | -30 | 34 | 1 | 0.366 | -108 | 34 | 10 | 0.002 | -106 | 39 | 7 | 0.006 | |
| ***random*** |  |  |  |  |  |  |  |  |  |  |  |  |  |  |  |  | |
| Year:com | 55 |  |  |  | 129 |  |  |  | 112 |  |  |  | 134 |  |  |  | |
| com | 209 |  |  |  | 83 |  |  |  | 135 |  |  |  | 148 |  |  |  | |
| block:Year |  |  |  |  | 111 |  |  |  |  |  |  |  |  |  |  |  | |
| block |  |  |  |  | 0 |  |  |  |  |  |  |  |  |  |  |  | |
| model |  |  |  |  |  |  |  |  | 217 |  |  |  | 202 |  |  |  | |
| Residual | 378 |  |  |  | 168 |  |  |  | 116 |  |  |  | 115 |  |  |  | |
| **(B)** |  |  |  |  |  |  |  |  |  |  |  |  |  |  |  |  | |
| (Intercept) | 318 | 65 | 10 | 0.001 | 363 | 34 | 70 | 0.000 | 317 | 14 | 178 | 0.000 | 320 | 14 | 175 | 0.000 | |
| SR | 33 | 10 | 9 | 0.002 | 40 | 21 | 4 | 0.059 | 31 | 9 | 12 | 0.001 | 35 | 9 | 15 | 0.000 | |
| *A. pratense* | -48 | 43 | 82 | 0.000 | -111 | 69 | 3 | 0.103 | -90 | 27 | 11 | 0.001 | -93 | 27 | 11 | 0.001 | |
| *A. sylvestris* | 0 | 43 | 83 | 0.000 | 150 | 81 | 4 | 0.061 | 117 | 26 | 19 | 0.000 | 68 | 28 | 6 | 0.015 | |
| *A. elatius* | 98 | 43 | 78 | 0.000 | 252 | 68 | 14 | 0.000 | 78 | 27 | 8 | 0.004 | 109 | 26 | 16 | 0.000 | |
| *D. glomeratis* | -23 | 43 | 83 | 0.000 | -70 | 72 | 1 | 0.329 | -27 | 28 | 1 | 0.327 | -2 | 29 | 0 | 0.948 | |
| *G. pratensis* | 56 | 43 | 82 | 0.000 | 6 | 72 | 0 | 0.931 | -66 | 27 | 6 | 0.017 | -71 | 28 | 6 | 0.011 | |
| *P. pratense* | -44 | 43 | 82 | 0.000 | -49 | 71 | 0 | 0.499 | 29 | 28 | 1 | 0.297 | 35 | 29 | 2 | 0.211 | |
| *P. trivialis* | 40 | 43 | 82 | 0.000 | -62 | 70 | 1 | 0.369 | -23 | 28 | 1 | 0.413 | -2 | 29 | 0 | 0.949 | |
| *T. pratense* | 35 | 43 | 83 | 0.000 | -17 | 72 | 0 | 0.809 | -65 | 28 | 6 | 0.018 | -67 | 28 | 6 | 0.017 | |
| *T. repens* | -114 | 43 | 76 | 0.000 | -66 | 75 | 1 | 0.372 | 47 | 28 | 3 | 0.090 | 22 | 29 | 1 | 0.435 | |
| ***random*** |  |  |  |  |  |  |  |  |  |  |  |  |  |  |  |  | |
| block | 0 |  |  |  |  |  |  |  | 85 |  |  |  | 95 |  |  |  | |
| block : Year | 179 |  |  |  |  |  |  |  |  |  |  |  |  |  |  |  | |
| com | 35 |  |  |  | 0 |  |  |  | 111 |  |  |  | 105 |  |  |  | |
| Year : com | 156 |  |  |  | 181 |  |  |  | 58 |  |  |  | 85 |  |  |  | |
| Residual |  |  |  |  | 462 |  |  |  | 167 |  |  |  | 153 |  |  |  | |
| **(C)** |  |  |  |  |  |  |  |  |  |  |  |  |  |  |  |  | |
| (Intercept) | 172 | 40 | 10 | 0.002 | 380 | 28 | 103 | 0.000 | 215 | 13 | 127 | 0.000 | 216 | 13 | 128 | 0.000 | |
| SR | 20 | 12 | 3 | 0.094 | 37 | 18 | 4 | 0.046 | 12 | 9 | 2 | 0.178 | 16 | 9 | 3 | 0.075 | |
| *A. pratense* | -33 | 38 | 21 | 0.000 | -122 | 57 | 5 | 0.032 | -95 | 27 | 12 | 0.001 | -95 | 27 | 12 | 0.000 | |
| *A. sylvestris* | 88 | 37 | 16 | 0.000 | 174 | 63 | 8 | 0.006 | 129 | 25 | 24 | 0.000 | 86 | 27 | 10 | 0.002 | |
| *A. elatius* | 144 | 36 | 6 | 0.016 | 249 | 53 | 21 | 0.000 | 67 | 28 | 6 | 0.016 | 89 | 27 | 11 | 0.001 | |
| *D. glomeratis* | 21 | 38 | 21 | 0.000 | 6 | 60 | 0 | 0.912 | -23 | 29 | 1 | 0.415 | 11 | 28 | 0 | 0.692 | |
| *G. pratensis* | 91 | 37 | 16 | 0.000 | -41 | 60 | 1 | 0.480 | -73 | 28 | 7 | 0.008 | -84 | 27 | 10 | 0.002 | |
| *P. pratense* | -41 | 38 | 20 | 0.000 | -83 | 59 | 2 | 0.154 | 21 | 29 | 1 | 0.463 | 29 | 28 | 1 | 0.306 | |
| *P. trivialis* | -25 | 38 | 21 | 0.000 | -57 | 59 | 1 | 0.324 | -15 | 29 | 0 | 0.599 | 10 | 28 | 0 | 0.718 | |
| *T. pratense* | -101 | 37 | 14 | 0.000 | -30 | 60 | 0 | 0.608 | -71 | 28 | 7 | 0.010 | -76 | 27 | 8 | 0.006 | |
| *T. repens* | -145 | 36 | 6 | 0.018 | -73 | 60 | 1 | 0.224 | 59 | 28 | 5 | 0.033 | 30 | 28 | 1 | 0.278 | |
| ***random*** |  |  |  |  |  |  |  |  |  |  |  |  |  |  |  |  | |
| block | 0 |  |  |  |  |  |  |  |  |  |  |  |  |  |  |  | |
| block : Year | 101 |  |  |  |  |  |  |  |  |  |  |  |  |  |  |  | |
| com | 114 |  |  |  | 150 |  |  |  | 114 |  |  |  | 108 |  |  |  | |
| Year : com | 147 |  |  |  | 174 |  |  |  | 54 |  |  |  | 73 |  |  |  | |
| Residual |  |  |  |  | 235 |  |  |  | 155 |  |  |  | 144 |  |  |  | |
| **(D)** |  |  |  |  |  |  |  |  |  |  |  |  |  |  |  |  | |
| (Intercept) | 146 | 33 | 8 | 0.004 | -13 | 15 | 1 | 0.389 | 102 | 3 | 223 | 0.000 | 105 | 4 | 212 | 0.000 | |
| SR | 13 | 9 | 2 | 0.136 | 0 | 9 | 0 | 0.956 | 19 | 1 | 135 | 0.000 | 19 | 1 | 101 | 0.000 | |
| *A. pratense* | -15 | 29 | 50 | 0.000 | 10 | 32 | 0 | 0.759 | 5 | 4 | 2 | 0.165 | 2 | 5 | 0 | 0.599 | |
| *A. sylvestris* | -89 | 28 | 40 | 0.000 | -39 | 38 | 1 | 0.302 | -13 | 4 | 13 | 0.000 | -18 | 4 | 17 | 0.000 | |
| *A. elatius* | -46 | 29 | 47 | 0.000 | 14 | 33 | 0 | 0.666 | 10 | 4 | 9 | 0.003 | 19 | 4 | 19 | 0.000 | |
| *D. glomeratis* | -44 | 29 | 48 | 0.000 | -89 | 33 | 7 | 0.007 | -4 | 4 | 1 | 0.223 | -13 | 4 | 8 | 0.004 | |
| *G. pratensis* | -35 | 29 | 48 | 0.000 | 37 | 33 | 1 | 0.258 | 7 | 4 | 4 | 0.036 | 13 | 4 | 9 | 0.003 | |
| *P. pratense* | -3 | 29 | 50 | 0.000 | 39 | 32 | 1 | 0.226 | 8 | 4 | 6 | 0.019 | 7 | 5 | 2 | 0.135 | |
| *P. trivialis* | 65 | 29 | 45 | 0.000 | 5 | 32 | 0 | 0.866 | -8 | 4 | 5 | 0.025 | -12 | 4 | 7 | 0.008 | |
| *T. pratense* | 136 | 26 | 24 | 0.000 | 14 | 33 | 0 | 0.678 | 6 | 4 | 3 | 0.080 | 9 | 5 | 4 | 0.042 | |
| *T. repens* | 30 | 29 | 49 | 0.000 | -5 | 35 | 0 | 0.897 | -12 | 4 | 12 | 0.000 | -8 | 5 | 3 | 0.074 | |
| ***random*** |  |  |  | 0.000 |  |  |  |  |  |  |  |  |  |  |  |  | |
| block | 0 |  |  | 0.011 |  |  |  |  |  |  |  |  |  |  |  |  | |
| block : Year | 85 |  |  | 0.009 |  |  |  |  |  |  |  |  |  |  |  |  | |
| com | 83 |  |  | 0.418 | 0 |  |  |  | 0 |  |  |  | 15 |  |  |  | |
| Year : com | 84 |  |  | 0.035 | 0 |  |  |  | 0 |  |  |  | 0 |  |  |  | |
| Residual |  |  |  | 0.000 | 257 |  |  |  | 92 |  |  |  | 82 |  |  |  | |

Table 2 Summary of linear mixed model analysis comparing observed and predicted species richness effects on community biomass, selection and complementarity effects. We fit random intercept models to test if community biomass (A), net biodiversity effects (B), selection effects (C) and complementarity effects (D) differ between experiments (current and pre-existing) and model predictions (PSF and Null). The fixed effect model data + SR + data : SR was tested against the random intercepts com and Year : com (interaction), where data represents the experiments/models being compared, SR = linear contrast of species richness, com = species composition, Year = Year of measurement (repeated measurements). Comparisons made: (I) pre-existing and current, (II) current and Null, (II) current and PSF, (IV) pre-existing and Null, (V) pre-existing and PSF and (VI) Null and PSF. Note that Intercept represents the group mean of the first group within data. If the interaction data : SR is insignificant, the species richness effect is similar between experiments/models being compared. Standard error (SE) are given in gm-2. Models were fit in R (R Core Development Team, 2015) with lmer (lme4, Bates et al., 2015), statistics were obtained with anova, Type III Analysis of Variance with Satterthwaite's method.

|  | **(I)** |  |  | **(II)** |  |  | **(III)** |  |  | **(IV)** |  |  | **(V)** |  |  | **(VI)** |  |  |
| --- | --- | --- | --- | --- | --- | --- | --- | --- | --- | --- | --- | --- | --- | --- | --- | --- | --- | --- |
|  | SE | *2* | *P* | SE | *2* | *P* | SE | *2* | *P* | SE | *2* | *P* | SE | *2* | *P* | SE | *2* | *P* |
| **(A)** |  | ** |  |  | ** |  |  | ** |  |  | ** |  |  | ** |  |  | ** |  |
| (Intercept) | 23 | 255 | 0 | 24 | 260 | 0 | 24 | 250 | 0 | 16 | 321 | 0 | 17 | 305 | 0 | 19 | 294 | 0 |
| data | 26 | 17 | 0 | 23 | 0 | 1 | 24 | 4 | 0 | 17 | 40 | 0 | 18 | 12 | 0 | 3 | 196 | 0 |
| SR | 13 | 20 | 0 | 14 | 18 | 0 | 14 | 18 | 0 | 9 | 33 | 0 | 9 | 31 | 0 | 11 | 24 | 0 |
| dara : SR | 17 | 1 | 0 | 15 | 1 | 0 | 16 | 0 | 0 | 11 | 0 | 1 | 12 | 0 | 1 | 2 | 3 | 0 |
| *Year : com* | *59* |  |  | *56* |  |  | *65* |  |  | *0* |  |  | *0* |  |  | *124* |  |  |
| *com* | *152* |  |  | *177* |  |  | *181* |  |  | *106* |  |  | *114* |  |  | *145* |  |  |
| *residual* | *264* |  |  | *233* |  |  | *238* |  |  | *169* |  |  | *177* |  |  | *27* |  |  |
| **(B)** |  |  |  |  |  |  |  |  |  |  |  |  |  |  |  |  |  |  |
| (Intercept) | 61 | 9 | 0 | 28 | 9 | 0 | 34 | 8 | 0 | 59 | 8 | 0 | 61 | 8 | 0 | 35 | 8 | 0 |
| data | 120 | 0 | 1 | 34 | 1 | 0 | 34 | 1 | 0 | 119 | 0 | 1 | 122 | 0 | 1 | 14 | 0 | 1 |
| SR | 13 | 10 | 0 | 12 | 11 | 0 | 12 | 12 | 0 | 6 | 26 | 0 | 6 | 29 | 0 | 5 | 49 | 0 |
| data : SR | 26 | 0 | 1 | 24 | 1 | 0 | 24 | 0 | 1 | 12 | 0 | 1 | 12 | 0 | 1 | 9 | 0 | 1 |
| *Year* | *116* |  |  | *38* |  |  | *48* |  |  | *117* |  |  | *120* |  |  | *48* |  |  |
| *residual* | *328* |  |  | *303* |  |  | *303* |  |  | *171* |  |  | *172* |  |  | *126* |  |  |
| **(C)** |  |  |  |  |  |  |  |  |  |  |  |  |  |  |  |  |  |  |
| (Intercept) | 61 | 7 | 0 | 13 | 12 | 0 | 13 | 11 | 0 | 40 | 8 | 0 | 42 | 7 | 0 | 30 | 7 | 0 |
| data | 74 | 4 | 0 | 25 | 35 | 0 | 25 | 35 | 0 | 77 | 0 | 1 | 80 | 0 | 1 | 13 | 0 | 1 |
| SR | 10 | 8 | 0 | 9 | 7 | 0 | 9 | 9 | 0 | 6 | 7 | 0 | 6 | 8 | 0 | 5 | 9 | 0 |
| data : SR | 20 | 1 | 0 | 18 | 3 | 0 | 18 | 2 | 0 | 12 | 0 | 1 | 12 | 0 | 1 | 9 | 0 | 1 |
| *Year* | *68* |  |  | *13* |  |  | *22* |  |  | *75* |  |  | *78* |  |  | *42* |  |  |
| *residual* | *253* |  |  | *224* |  |  | *225* |  |  | *168* |  |  | *168* |  |  | *125* |  |  |
| **(D)** |  |  |  |  |  |  |  |  |  |  |  |  |  |  |  |  |  |  |
| (Intercept) | 48 | 2 | 0 | 21 | 3 | 0 | 23 | 2 | 0 | 24 | 8 | 0 | 24 | 8 | 0 | 4 | 11 | 0 |
| data | 54 | 5 | 0 | 16 | 52 | 0 | 16 | 54 | 0 | 42 | 1 | 0 | 43 | 1 | 0 | 4 | 1 | 0 |
| SR | 7 | 3 | 0 | 6 | 6 | 0 | 6 | 7 | 0 | 4 | 16 | 0 | 4 | 16 | 0 | 1 | 398 | 0 |
| data : SR | 15 | 0 | 1 | 12 | 1 | 0 | 12 | 1 | 0 | 8 | 0 | 1 | 8 | 1 | 0 | 1 | 0 | 1 |
| *Year* | *51* |  |  | *20* |  |  | *23* |  |  | *41* |  |  | *41* |  |  | *6* |  |  |
| *residual* | *185* |  |  | *146* |  |  | *146* |  |  | *112* |  |  | *112* |  |  | *20* |  |  |

**Table 3** Correlation Analysis of Observed and Predicted Community and Species Biomass. We performed simple regression analysis in R (R Core Development Team, 2015) to evaluate the correlation between predictions of PSF and Null models for community biomass **(a)** and species biomass **(b)** with observations from the current (2016-2017) and pre-existing (2003-2004) experiment. Slopes and corresponding 95% confidence intervals (CI, 1 = 2.25 %; 2 = 97.25 %) are reported, as well as correlation coefficients (R2), AIC, and deviance to evaluate ‘goodness’ of fit.

|  | model | experiment | slope | CI(1) | CI(2) | DF res | R2 | AIC | deviance |
| --- | --- | --- | --- | --- | --- | --- | --- | --- | --- |
| **(a)** | *Community biomass* | | |  |  |  |  |  |  |
|  | Null | current | 1.087 | 0.855 | 1.319 | 198 | 0.301 | 2892 | 21605205 |
|  | PSF | current | 0.933 | 0.722 | 1.145 | 198 | 0.276 | 2899 | 22363958 |
|  | Null | pre-existing | 0.335 | 0.179 | 0.491 | 198 | 0.083 | 2733 | 9754292 |
|  | PSF | pre-existing | 0.284 | 0.144 | 0.425 | 198 | 0.075 | 2734 | 9842454 |
| **(b)** | *Species biomass* | | |  |  |  |  |  |  |
|  | Null | current | 1.039 | 0.942 | 1.135 | 610 | 0.421 | 8739 | 56421262 |
|  | PSF | current | 1.032 | 0.932 | 1.131 | 610 | 0.404 | 8757 | 58119961 |
|  | Null | pre-existing | 0.631 | 0.567 | 0.695 | 610 | 0.382 | 8230 | 24560238 |
|  | PSF | pre-existing | 0.625 | 0.559 | 0.691 | 610 | 0.364 | 8247 | 25268967 |

**Table 4** Observed and Predicted Relative Species Biomass. Relative species biomass (con) was estimated for observation of the **(a)** pre-existing (2003-2004) and **(b)** current experiment (2016-2017) and predictions of **(c)** Null and **(d)** PSF models. Corresponding 95% confidence intervals (CI, 1 = 2.25 %; 2 = 97.25 %) and standard deviation (SD) are reported.

| data | species | con | N | SD | CI(1) | CI(2) |
| --- | --- | --- | --- | --- | --- | --- |
| **(a)** | *A. pratense* | 0.391 | 148 | 0.338 | 0.34 | 0.45 |
|  | *A. sylvestris* | 0.065 | 148 | 0.207 | 0.03 | 0.10 |
|  | *A. elatius* | 0.728 | 148 | 0.190 | 0.70 | 0.76 |
|  | *D. glomeratis* | 0.556 | 148 | 0.324 | 0.50 | 0.61 |
|  | *G. pratensis* | 0.104 | 148 | 0.246 | 0.06 | 0.14 |
|  | *P. pratense* | 0.371 | 148 | 0.324 | 0.32 | 0.42 |
|  | *P. trivialis* | 0.148 | 148 | 0.240 | 0.11 | 0.19 |
|  | *T. pratense* | 0.293 | 148 | 0.273 | 0.25 | 0.34 |
|  | *T. repens* | 0.129 | 148 | 0.246 | 0.09 | 0.17 |
| **(b)** | *A. pratense* | 0.317 | 186 | 0.364 | 0.26 | 0.37 |
|  | *A. sylvestris* | 0.005 | 193 | 0.073 | 0.00 | 0.02 |
|  | *A. elatius* | 0.890 | 154 | 0.152 | 0.87 | 0.91 |
|  | *D. glomeratis* | 0.590 | 169 | 0.373 | 0.53 | 0.65 |
|  | *G. pratensis* | 0.139 | 162 | 0.298 | 0.09 | 0.18 |
|  | *P. pratense* | 0.395 | 170 | 0.396 | 0.33 | 0.45 |
|  | *P. trivialis* | 0.104 | 154 | 0.258 | 0.06 | 0.14 |
|  | *T. pratense* | 0.109 | 178 | 0.272 | 0.07 | 0.15 |
|  | *T. repens* | 0.119 | 161 | 0.278 | 0.08 | 0.16 |
| **(c)** | *A. pratense* | 0.320 | 1224 | 0.273 | 0.30 | 0.34 |
|  | *A. sylvestris* | 0.093 | 1224 | 0.211 | 0.08 | 0.10 |
|  | *A. elatius* | 0.672 | 1224 | 0.198 | 0.66 | 0.68 |
|  | *D. glomeratis* | 0.244 | 1224 | 0.266 | 0.23 | 0.26 |
|  | *G. pratensis* | 0.407 | 1224 | 0.280 | 0.39 | 0.42 |
|  | *P. pratense* | 0.603 | 1224 | 0.235 | 0.59 | 0.62 |
|  | *P. trivialis* | 0.177 | 1224 | 0.244 | 0.16 | 0.19 |
|  | *T. pratense* | 0.299 | 1224 | 0.277 | 0.28 | 0.31 |
|  | *T. repens* | 0.126 | 1224 | 0.229 | 0.11 | 0.14 |
| **(d)** | *A. pratense* | 0.328 | 1224 | 0.278 | 0.31 | 0.34 |
|  | *A. sylvestris* | 0.082 | 1224 | 0.206 | 0.07 | 0.09 |
|  | *A. elatius* | 0.693 | 1224 | 0.190 | 0.68 | 0.70 |
|  | *D. glomeratis* | 0.202 | 1224 | 0.266 | 0.19 | 0.22 |
|  | *G. pratensis* | 0.405 | 1224 | 0.284 | 0.39 | 0.42 |
|  | *P. pratense* | 0.597 | 1224 | 0.247 | 0.58 | 0.61 |
|  | *P. trivialis* | 0.153 | 1224 | 0.240 | 0.14 | 0.17 |
|  | *T. pratense* | 0.333 | 1224 | 0.285 | 0.32 | 0.35 |
|  | *T. repens* | 0.148 | 1224 | 0.240 | 0.13 | 0.16 |
